# The Importance of Water Typologies in Lay Entomologies of *Aedes aegypti* Habitat, Breeding and Dengue Risk: A Study from Northern Australia

**DOI:** 10.3390/tropicalmed3020067

**Published:** 2018-06-18

**Authors:** Darlene McNaughton, Emma R. Miller, George Tsourtos

**Affiliations:** College of Medicine and Public Health, Flinders University, GPO Box 2100, Adelaide 5001, Australia; emma.miller@flinders.edu.au (E.R.M.); George.Tsourtos@flinders.edu.au (G.T.)

**Keywords:** lay knowledge, dengue fever, lay entomologies, Australia, medical anthropology

## Abstract

Dengue fever is making a significant comeback globally and its control still depends largely on residents’ actions. Community awareness and education are central to its management; however, programmes have had limited impact, because they are often based on short-term research and limited awareness of the socio-ecological contexts wherein local knowledge of dengue and its vectors (lay entomology) is produced and enacted in and through place. Long-term studies of lay knowledge of dengue vectors are very rare, even though they are essential to the development of effective, targeted community education campaigns and mobilisation. In this paper, we examine the popular belief that dengue vector, *Aedes aegypti,* is ubiquitous in the north Australian landscape and demonstrate how local typologies of water are central to the reasoning underwriting this assumption. We show how these logics are fortified by people’s lived experiences of mosquitoes and the watery abodes they are thought to reside in, as well as through key messages from health education. We posit that long term, context-sensitive research approaches are better able to identify, understand and later address and challenge assumptions and may be more effective at informing, empowering and mobilizing the public to combat dengue fever.

## 1. Introduction

Interactions and relationships between mosquitoes, dengue viruses, and human beings have a long, dynamic, and complex history. Recent decades bore witness to a significant increase in the incidence of dengue fever worldwide, which was recognised as the ‘…most important arboviral disease of humans’ [1]. More than 40% of the world’s population is deemed to be at risk from the virus, which is now endemic in more than 100 countries [1,2,3,4,5].

While dengue viruses are transported across regions and between countries by human beings, subsequently being contracted and transmitted by female mosquito vectors to other humans, it is not only the viruses that are on the move. In these human–insect entanglements, mosquito vectors are also appearing in new places, or returning to regions previously deemed free of both the vectors and the disease, including northeast Australia, the site of this study.

Outbreaks of dengue fever were first reported in northeast Australia in 1879, and by the early decades of the 20th century, outbreaks of dengue were recorded as far south as Sydney, located 2500 km away, in the southeast of the country. The incidence of dengue began changing in the 1960s, when the use of residual insecticides (including DDT), and the introduction of reticulated water became more widespread, leading to a decrease in both outbreaks and vector populations [6,7,8,9,10,11]. For the next 26 years, there were no recorded incidents of dengue fever in Australia until 1981, following the establishment of an international airport, where an outbreak was recorded in the city of Cairns in northern Australia [12,13,14]. Since that time, there have been four fatalities and 63 outbreaks in the region—covering an area from the Torres Strait Islands in the far north, to the city of Townsville more than 1000 km to the south. Although the virus is not currently endemic in northern Australia, all four dengue viruses were recorded in human beings returning to or arriving in the region. Over the last 15 years, outbreaks of dengue fever became a common, usually annual, occurrence in northeastern Australia, coinciding with the monsoon or wet season.

At present, the *Aedes aegypti* mosquito is the primary vector of dengue fever in northern Queensland, and is known locally as the ‘dengue mosquito’ or ‘dengue mozzie’. *Aedes albopictus* was implicated as a second vector of dengue; however, it is currently confined to areas located within the Torres Strait region of northern Queensland, and outside of the study area of this paper. *Ae. aegypti* is a highly domesticated (peri-urban) species that lays eggs on and in containers holding fresh water (i.e., tap and/or rain water), located in and around homes and other buildings in suburban areas (e.g., offices, construction sites, vases in cemeteries, and some types of subterranean pits) [15]. The most common breeding sites are containers with straight edges, on which the female mosquito lays her eggs. These include items such as vehicle tyres, potted plant bases, vases, plastic tarpaulins, boats, blocked roof gutters, buckets, pet bowls, watering cans, and fallen palm tree fronds [15,16].

*Ae. aegypti* is not found and does not breed in creeks, swamps, lagoons, rivers, or puddles of water, which are common features in the regional landscape. Research in this region also suggested that they do not fly far (100–200 m at most) from humans, their preferred source of blood, and that they bite mostly during the day [17]. In other words, *Ae. aegypti* mosquitoes live with us, and we with them. They are born, live, and die in and around our homes, and places of work and leisure. The buildings we make provide cool places to rest—on walls, in wardrobes, in gardens, and under furniture—and it is from these places that the female mosquitoes venture out to secure a blood meal from us, and at times, from a resident dog or chicken. This blood provides the protein needed to produce her eggs, which she will deposit in and on the aforementioned containers we have so thoughtfully provided. In some cases, we also provide the fresh water for these containers as well (vases and potted plant bases); in other cases, it is the rain or the garden hose that provides the fresh water her eggs will be submerged in, so that the lifecycle of the mosquito can begin.

This interspecies entanglement, while longstanding and complex, is also heavily reliant on humans providing the environments in which mosquitoes can thrive, and through the inadvertent seeking of a blood meal, contract and transmit dengue between people and other *Ae. aegypti* mosquitoes. As such, the prevention of dengue fever in this region, like most others, centres on reducing human exposure to the mosquito vector through five key measures: the removal of breeding sites, the use of insecticide by local authorities and householders, the application of personal insect repellent, insect-screened accommodation, and ongoing community education campaigns. During the period of this study and into the present day, local health authorities also employed dedicated teams who used surveillance and insecticide, and initiated the removal of breeding sites on a property-by-property basis, in an effort to reduce risk and respond to outbreaks. As is the case internationally, protection and prevention also rely, in part, upon residents’ actions and public knowledge of the mosquito, the disease, and its transmission.

Since the return of dengue fever to the region in the 1980s, public education was at the forefront of dengue prevention campaigns, alerting residents to the risks of the disease and promoting preventive measures, especially the identification and removal of potential breeding containers from around suburban homes. The central message across these previous campaigns was simple: stop the dengue mosquitoes from breeding in and around your home, and you can protect yourself and your family, and stop the disease from spreading. These campaigns also attempted highlighting the link between *Ae. aegypti* (including a focus on its appearance and behaviour) and the disease. As such, they encouraged residents to be more observant of the mosquitoes that bite them, and even encouraged them to identify the mosquitoes. These campaigns consistently described the *Ae. aegypti* mosquito as a dark-coloured mosquito with black and white stripes on its legs and body/abdomen, and also used images of the vector. In short, the focus of these campaigns was on the actions and responsibility of domiciled residents to recognise the vector and potential breeding sites, and to remove the latter, namely containers holding fresh water. This resulted in some success. In the ethnographic interviews undertaken by the first author from 2007–2008, and in subsequent and extensive social research in 2008–2011 with hundreds of local residents, *Ae. aegypti* was consistently described as a black and white striped mosquito, and mosquitoes with this general appearance were associated with dengue—with the appellation ‘dengue mosquito’ being the most common form of reference to *Ae. aegypti* in local parlance [18,19]. This strongly suggests that many residents do connect mosquitoes of a certain appearance with the disease [12,13]. The problem, of course, is that many of the 300 mosquito species in the region, including nuisance mosquitoes, have black or dark grey striped legs and/or abdomens, including those found in locales beyond the domestic environment (e.g., *Aedes notoscriptus* and *Aedes vigilax*). Identifying the mosquito via image and description campaigns appears to have inadvertently reinforced ideas about the ubiquity of ‘dengue mosquitoes’ in the landscape, as we previously showed [18].

It is well established that many health interventions fail because they are designed based on a limited awareness of the lay understandings of a disease, its transmission and vectors, and the socio-political and historical contexts in which this knowledge was produced and enacted [20,21,22,23]. Several ground-breaking studies into the lay understandings of malaria and its vectors demonstrated that it is essential to know how the disease and its vector are understood if we are to communicate effectively, and reduce the incidence of disease [24,25,26,27,28,29]. However, few published accounts examined the lay knowledge of dengue fever in any depth (see, for example, references [19,30,31,32,33,34,35,36,37,38,39]), with even fewer studies exploring the lay understandings of dengue vectors [19,24,33,36,38,40], or the assumptions supporting lay knowledge. Furthermore, studies on dengue and lay knowledge are also rare in so-called developed countries [41,42].

In recognition of these gaps in the extant literature, in 2007–2008, McNaughton undertook long-term ethnographic research into the lay understandings of the dengue virus, its transmission, and its primary vector, *Ae. aegypti*, in northern Australia, which was followed up with an extensive program of qualitative and quantitative research and engagement. Using an ethnographic research design that included participant observation with residents and mosquito-control staff, focus groups (*n* = 20), and in-depth semi-structured interviews (*n* = 50) with Cairns residents, three key findings emerged (reported elsewhere [18,41,42]). Firstly, many residents described the ‘dengue mosquito’ as being dark-coloured and visibly striped, and believed that *Ae*. *aegypti* mosquitoes were ubiquitous in the landscape, living and breeding around the home or other humanised buildings, and in a variety of geographical spaces located beyond them. Secondly, while most residents were able to accurately identify key breeding containers, many also spontaneously suggested that the ‘dengue mosquito’ breeds in ‘water that is lying around’. This included pools of water, puddles, swamps, lagoons, ponds, creeks, beaches, mangroves, and in organic debris such as plants and palm fronds located in forested areas. Notably, these were often sites located out of their immediate control or responsibility. When asked to ‘describe what kind of water the mosquito breeds in’, residents referred to ‘water that is lying around’, ’stagnant water’ (described further as still water, or water that had ‘gone off’ and was ‘undrinkable’), and ‘dirty water’ (described further as undrinkable, full of debris, ‘discoloured water’, ‘murky’, or ‘not clear’). In a small number of cases, ‘brackish water’ was also included in the responses, and was often described as slightly salty, undrinkable water. Residents also appeared to assume that *Ae. aegypti* ‘breeds in water’, laying its eggs on or in the water, when its preference is actually to lay eggs on the sides of containers. Although people responded individually to these questions, their responses were strikingly consistent, especially in the connection they made between ‘water lying around’, and the presence and prevalence of the vector [18].

Although these understandings were at odds with entomological knowledge, residents appeared to draw on their actual experiences of mosquitoes, often describing being bitten by (and squashing) dark-coloured, striped ‘dengue mosquitoes’ in a variety of places, and reinforcing key information from past education campaigns [18]. Indeed, a common sentiment among Cairns residents was that, no matter how much effort they made as individuals to remove breeding sites, etc., this mosquito would continue entering their homes, workplaces, and backyards from elsewhere in the landscape. For some, this manifested in calls for state and local governments to ‘do more’, and showed that many residents experienced limited control over the situation, and even hopelessness.

These findings might read as an indication of the nature and scope of public ‘ignorance’ surrounding the disease and its vector, something best addressed through further education. However, from an anthropological perspective, this is not how it would be understood, in part because the shared nature and recurrence of these understandings suggested much more. These results indicated that deeper, taken-for-granted assumptions were at work, and were worthy of further exploration and contextualisation. This paper grew out of these findings. It had two central aims: to contribute to the significant gap in current knowledge around the lay understandings of vectors, and to explore these understandings further through the examination of a larger study and sample of the northern Queensland residents’ knowledge of the breeding sites and habitats of *Ae. aegypti* mosquitoes.

Therefore, our aim was not to restate earlier findings, but to contextualise and examine them in more depth through a detailed reanalysis of existing public-health telephone-survey data (including data not previously published), which were previously undertaken during and after the ethnographic study. Thus, in this paper, we attempted making the lay knowledge of *Ae. aegypti* more ‘culturally readable’ by exploring the following questions: (1) What do northern Queensland residents in the two dengue-affected cities of Cairns and Townsville know about the breeding sites and habitat of the *Ae. aegypti* mosquito? Furthermore, more specifically, (2) what logic gives rise to these understandings?

## 2. Materials and Methods

The data we reanalysed came from three waves of public health surveys conducted in 2004, 2007, and 2008 by Queensland Health in the cities of Townsville and Cairns. These are the largest cities in tropical northern Australia. They both have distinct dry and wet seasons, with mosquitoes being particularly abundant, thus more visible, during the wetter months (November to March). Many residential suburbs in both cities are located near beaches, swamps, rivers, and creeks, and both cities have highly mobile and growing populations [43].

To briefly summarise the methodology of the surveys, the sampling frame was the electronic white pages (EWP) telephone directory, which gives addresses for households with a landline telephone. Two hundred households were surveyed in each city in each of the three survey periods, and census data were used to establish broad quotas of respondents based on age and gender. Respondents were chosen through a random selection of numbers, with visitors to the region excluded from each survey.

The content of the surveys was developed at the Queensland Health’s Tropical Population Health Services in Cairns. Using a focus group with key stakeholders and mosquito-control experts (Schmidt 2008, pers. comm.), key staff arrived at a consensus on the questionnaire design, especially regarding the principal environments where the public might see *Ae. aegypti* mosquito breeding places. The main domains of the questionnaire focused on the respondents’ lay knowledge regarding correct and incorrect breeding sites. Questions also covered the respondents’ education, age, gender, and length of residence in northern Queensland. Results from most of the questions featured in the survey are not yet published.

Questions were asked about the ‘dengue mosquito’ (*Ae. aegypti*) breeding sites. In the 2004 and 2007 surveys, respondents were asked, ‘Where do you think dengue mosquitoes breed?’ Responses were recorded, and the participants were then prompted about whether they believed dengue mosquitoes breed in various environments, where options 1, 2, 3, and 8 represented incorrect breeding sites, and the other options represented correct breeding sites. These environments include drivers (i.e., freshwater and/or estuarine water courses that can expand in size and volume depending on seasonal rainfall levels), swamps (shallow, land-locked freshwater bodies with moderate to dense vegetation), drains (a network of high-volume drains for transporting large volumes of storm water), household containers, vases (for displaying cut flowers or striking plant cuttings; a potential breeding site for *Ae. aegypti*), rainwater tanks (used for the storage of rainwater; a potential breeding site for *Ae. aegypti*), roof gutters (located on domestic and industrial buildings; when blocked, a potential breeding site for *Ae. aegypti*), saltwater (brackish or saltwater swamps, shallow water bodies with moderate tolerance to salinity, periodically influenced by tidal flows from estuarine waters), tyres (discarded vehicle tyres), and lastly, pot-plant bases (bases of plant pots).

A new series of questions was added to the 2008 survey to explore the local knowledge of breeding sites in relation to types of water. It read as follows: ‘I am going to read out some places where dengue mosquitoes might breed. For each one, please tell me if you think that dengue mosquitoes can or cannot breed in that place.’ The given options are listed below.

Water clean enough to drink
Water that is reasonably clean
In dirty water that you would not drink
Stagnant/still/stale water/water lying around
Fresh water

Four hundred participants completed the survey in each of the three years, and their demographic data and other details are presented in Table 1. Almost 50% of all respondents in each year were female, and the median age was in the 36- to 45-year-old age group. In total, 60% of participants reported residing in northern Queensland for ‘most’ of their lives, although this differed according to year, with statistically fewer residents in 2007 falling into this group (51%, *p* < 0.01 relative to both previous survey years). Nonetheless, the median duration of residence among the remaining participants was 10 years (interquartile range: 4–15 years). Around 69% of respondents were employed, either part time or full time, and 73% were buying or already owned their own home. Household size was similar across all survey years, with a median of three members.

### Secondary Analysis of Data

Data were analysed using Stata software (release 13, Stata Corporation, College Station, TX, USA). There were no missing data, so all observations were included in the analysis. In the original surveys, data were collected by quota to ensure that all demographic groups were represented in the sample. The recruitment method also ensured there were no statistical differences in age and sex among participants from Cairns or Townsville, and chi-square and Mann–Whitney U tests were conducted after recruitment demonstrated there were no significant differences (*p* > 0.05) between the two cities for employment status and length of residence in northern Queensland. For this reason, data from the two cities were combined for all analyses.

In the secondary analysis, we developed total scores for correctly identified mosquito breeding sites, weighted according to the different numbers of options presented to participants in each year (11 in 2004, 13 in 2007, and 16 in 2008). We combined unprompted and prompted responses for 2004 and 2007. This ‘breeding site score’ formed the main dependent variable for bivariate and multivariate analyses against a range of socio-demographic data, including the year of the survey, and exposure to television and radio advertising prior to the survey. Our bivariate analysis included chi-square tests and other nonparametric techniques. We built log-binomial and regression models to identify independent predictors of breeding site scores for each year of the survey. All data were tested using a significance level of 0.05.

## 3. Results

The respondents’ knowledge of *Ae. aegypti* breeding sites was analysed in terms of choosing the correct survey options (i.e., household containers, vases, rainwater tanks, roof gutters, tyres, and pot-plant bases), and not choosing the incorrect options (i.e., rivers, freshwater swamps, drains, and saltwater swamps). A weighted score (maximum 1.0) was developed to account for correct responses as a proportion of the options offered. The median score for 2004 was 0.82 (interquartile range: 0.73–0.91), indicating that 82% of responses made by participants were correct. Scores decreased over the next two years, with scores of 0.77 (interquartile range: 0.62–0.85) and 0.69 (interquartile range: 0.63–0.81) recorded in 2007 and 2008, respectively. A Kruskal–Wallis H test score for the three years demonstrated that there was a statistically significant difference in overall breeding site score (χ^2^ = 65.781, *p* < 0.001).

For each of the three surveys, bivariate nonparametric tests were undertaken to test the associations between breeding site score and various demographic variables (see Table 2). Males had significantly higher scores for all years. Full- and part-time workers had significantly higher scores overall than those not in paid employment, and also for the years 2007 and 2008. There was no difference in median scores according to employment status in 2004. Relative to renters, those who owned or were buying their homes had higher scores in 2008 (but not in 2004 or 2007). Excluding those reporting ‘lifelong’ residence in northern Queensland, residence for shorter than the median time of 10 years was associated with lower scores in 2008, but not in other years. Age, lifelong northern Queensland residence, and household size were not associated with breeding site score for any of the study periods.

### 3.1. Freshwater Quality Selection

Additional breeding site items focusing on water quality were included in the 2008 survey, three of which related to freshwater options as suitable aquatic environments for *Ae. aegypti* breeding: ‘water clean enough to drink’, ‘water that is reasonably clean’, and ‘fresh water’. The remaining two additional options were ‘dirty water that you would not drink’ and ‘stagnant or stale water lying around’. Two hundred and forty-four respondents (61%) correctly identified all three freshwater options as breeding sites. However, only two respondents (0.5%) were correct about all five options as potential breeding sites, and 25 respondents (6.3%) were incorrect about all five options. A score out of five correct was strongly correlated with total breeding site score (Spearman’s rho = 0.63, *p* < 0.001).

### 3.2. Multivariate Analyses

We built binary regression models for the outcomes above and below median breeding site score for each year. Outcomes were expressed as ratios for achieving median or above scores, and all models were also age-adjusted. Binary variables for gender, employment status, and home ownership were modelled for each year. Employment status was not significant in any model, and as such, was dropped out of the analysis. Males independently predicted higher breeding site scores in 2007 and in 2008 (score ratio 1.4), as did home ownership in 2008. Males were 1.5 times more likely than females to score median or above in 2007, and 1.3 times more likely in 2008. Home ownership predicted higher scores in 2004 and 2008, with home owners 1.3 times more likely to score median or above than renters in 2004, and 1.4 times more likely in 2008. The greatest absolute difference in score ratio was for male sex in 2007 (22%), with other absolute differences ranging from 13% to 15%. Exposure to radio and television advertisements was added to the 2007 and 2008 models, but remained insignificant once adjusted for gender and home ownership. The outcomes for the final binary models are presented in Table 3.

All potential factors were also included in regression models using breeding site score as a continuous variable with no further predictors identified; however, slight variations in either sex or home ownership emerged as independent variables in the first survey. In 2004, only male sex was significant (adjusted for age and home ownership)—β = 0.042, t(3) = 2.85, *p* = 0.005. In 2007, (similar to the log-binomial model) only male sex was again significant (adjusted for age and home ownership)—β = 0.063, t(3) = 4.55, *p* < 0.001. Both male sex and home ownership significantly predicted breeding site scores in 2008—β = 0.031, t(3) = 2.60, *p* = 0.010, and β = 0.057, t(3) = 3.96, *p* < 0.001, respectively.

## 4. Discussion

Our secondary analysis of the three surveys suggested that few people were able to identify key breeding sites correctly, or rejected all incorrect breeding sites. Although prompting slightly improved this, most respondents still chose both correct and incorrect breeding sites, with the latter located, for the most part, in the broader landscape outside the domestic environs and the individual’s control. These results supported the earlier hypothesis that many residents assume *Ae. aegypti* inhabits and breeds in a variety of places in the landscape, both within and beyond their homes [18]. The results also demonstrated the salience of water in local understandings. In particular, our second hypothesis (residents believe dengue mosquitoes breed in certain types of water) appeared to give rise to the lay understandings of the ubiquity of these insects in the landscape, adding something new to our earlier work. Further data analysis revealed significant differences within the populations surveyed, with more male and employed respondents identifying correct responses than female and non-employed respondents. However, the results clearly demonstrated that most people assume *Ae. aegypti* breeds in a variety of watery places, both within their personal control and beyond it in the wider landscape.

Another important finding came from responses to the open-ended question, ’Where do you think dengue mosquitoes breed?’, in the 2004 and 2007 surveys. The most frequent response to the question fell into the category of ‘water lying around’ in the landscape. Moreover, the percentage of respondents who identified ‘water lying around’ as a key breeding site increased over time, from 48% in 2004 to 74% in the 2007 survey. These surveys did not follow the same cohort, but the size of the increase was marked, and suggested that this belief may have intensified over this period. There were annual dengue outbreaks in these years, as well as active education and prevention campaigns.

In the 2008 survey, respondents were asked an additional question about the types of water *Ae. aegypti* might breed in. Only two respondents (out of 400) rejected all three incorrect answers: water you would not drink, stagnant/stale, and brackish water. While respondents commonly selected ‘fresh water’ and ‘water clean enough to drink’ as breeding sites, more than 90% of those surveyed chose at least two of the other water types—‘*dirty water that you would not drink*’ and/or ‘*stagnant/still/stale water/water lying around*’. Although significant associations were found for gender and employment status, it was evident that while most respondents associated fresh (rather than salty or brackish) water with *Ae. aegypti*, they also linked it to water that is still, stagnant, stale, and lying around in the landscape. Moreover, there were no significant correlations in the 2008 survey period between demographic variables and respondents’ ability to correctly reject dirty water breeding sites.

These results confirmed that water sitting in the landscape and in containers was very prominent in the lay understandings of breeding sites (see also Reference [18]). The imagery of the phrase ‘water lying around’ implies immobility, stillness, and stagnation. It also suggested that for residents of both cities, the abundance of water sitting in the landscape produces breeding sites, *Ae. aegypti* mosquitoes, and potentially dengue. This view was widespread and we argue that it is linked to a broader logic giving rise to the lay understandings—namely, that dengue mosquitoes are ubiquitous, and breed in a variety of locales and types of water. For some, the logic seems to proceed as subsequently described. The dengue mosquito (a dark-coloured mosquito with black and white stripes on its legs and body) is experienced (biting behaviour) and seen (other similar mosquitoes) in many places beyond the domestic environment; it lays its eggs in or on water that is abundant and ‘lying around’ the landscape (and the home/yard) during the wet season, including in household containers, gutters, tyres, ponds, puddles, creeks, etc.; thus, the dengue mosquito is ubiquitous in the landscape. This new finding not only added to our earlier study, but supported the results of the ethnographic research from which this study’s hypotheses were developed.

### Implications

In trying to unpack and contextualise the lay knowledge about *Ae. aegypti*, it was clear that the observations and experiences of the local environment were key factors in the development of local understandings—something rarely addressed in the extant dengue literature [38], but widely acknowledged in medical anthropology and sociology [20]. During the monsoon or wet season, as it is locally known (November to March), water is an abundant, persistent, and highly-visible feature of the landscape in tropical northern Queensland. Rain falls frequently, sometimes relentlessly, in significant quantities. Water blocks roads and pathways, fills swamps, creeks, gullies, low-lying land, paddocks, ponds, sports fields, and residents’ backyards, forming new bodies of water, and reinvigorating old ones. In the wet season, in both cities, water commonly remains in situ for days or weeks at a time, and occupies large parts of the landscape.

Coinciding with this watery abundance, the wet season is the period when mosquito populations increase significantly. Mosquitoes of all types become a more visible and felt presence at this time because of increased population density, mating, and biting behaviour. It is, therefore, highly likely that the strong association of ‘dengue mosquitoes’ with both fresh and dirty water was fortified, in part, by people’s broader experiences of mosquitoes in this particular region, especially during the wet season. For example, *Aedes notoscriptus* is diurnal in its blood feeding, exhibits similar behaviour to *Ae. aegypti*, and it also breeds in fresh water in containers around the home and garden. Significantly, several mosquitoes bearing a striking resemblance to *Ae. aegypti* also breed in water bodies located outside the home. These include *Ae. notoscriptus,* which breeds in fresh water in rock pools and tree holes located near freshwater swamps, *Culex annulirostris,* which prefers freshwater swamps, *Aedes vigilax* in salt marshes, and *Verralina funerea* in brackish swamps. Given the complexity of the local mosquito population, the similarities in the appearances of many species, and the difficulty of identifying mosquitoes with the naked or untrained eye, it was not surprising that local residents thought *Ae. aegypti* mosquitoes were biting them day and night in a variety of locales, including fresh and dirty or stagnant water.

This has important implications for future education, prevention initiatives, policy, and public health. This study showed that significant numbers of residents associated ’dengue mosquitoes’ with rivers, creeks, swamps, and beaches, as well as with fresh and stale water within their domestic environment. Many of these areas are beyond their immediate control and responsibility. The similarity in the pattern of responses across the three samples and between cities also suggested that the lay knowledge about *Ae. aegypti* breeding habits and habitats changed little over time, and possibly became more entrenched in some aspects. Thus, while residents accepted certain key messages from local health campaigns, they appeared to have added the information to earlier or alternative understandings and experiential knowledge, including the idea that dengue mosquitoes are ubiquitous and breed anywhere there is water lying around, especially water that is discoloured, dirty, or undrinkable.

These findings also provided insights into possible future directions for public health campaigns, which might include greater emphasis on the following messages:

1. *Ae. aegypti* live with or near people because female mosquitoes require a blood meal to produce their eggs, and humans are their preferred source.
2. *Ae. aegypti* are not able to fly far, at most around 100–200 m; so, if they are present in the home, then it is likely they are breeding nearby.
3. *Ae. aegypti* is a container breeder—they lay their eggs at the edges of containers, unlike other mosquitoes that commonly lay their eggs in or on the water.
4. There are many mosquitoes in the swamps, creeks, and beaches that look very similar to, but are not *Ae. aegypti*.

These insights could be used to produce more targeted education campaigns, tailored to populations and contexts, which, as many key commentators in the field of health education and communication showed, are essential to improving the impact and success of such campaigns [44,45].

There is a need for a study drawing on anthropological results, and using a longitudinal-repeated-measures design, in which a cohort of respondents completes a questionnaire several times at critical junctures, such as before, during, and after major public health campaigns on how to prevent contracting dengue fever. This is a strong design that can produce meaningful results regarding the extent to which a change in phenomenon occurred [46]. However, a large sample size would be needed because of an anticipated high attrition rate. According to the Australian Census (Australian Bureau of Statistics 2006), Cairns and Townsville have highly mobile populations, with more than 40% of respondents not living in the region five years earlier.

## 5. Conclusions

Even if new programs for vaccines, antiviral drugs, genetic modification, or biological control are successful in the future, for now, residents will continue to be called upon to undertake protective measures against vectors, and to reduce the potential for disease transmission. We learned from malaria studies that, although long-term anthropological studies into the lay knowledge of mosquito vectors are essential to the development of more targeted, effective education and mobilisation internationally, such context-sensitive studies are still rare in dengue research, and this represents a significant gap in knowledge.

In this paper, we examined the widely held view that *Ae. aegypti* is ubiquitous in the northern Queensland landscape, confirming earlier findings and demonstrating new ones—local typologies of water are central to the logic giving rise to this assumption. We showed that these assumptions were widespread across local populations, fortified by people’s experiences of mosquitoes in the landscape, especially dark-coloured, striped mosquitoes, and reinforced though the uptake of key messages from health campaigns. This logic also provided new context-sensitive insights into the lay knowledge in this region, which may be of use for future, more targeted education campaigns, which, as key commentators in the health education and communication field demonstrated, are key to improving the impact of these initiatives [45,46].

## Figures and Tables

**Table 1:** Participant characteristics in northern Queensland—2004, 2007, and 2008.

Characteristic	2004	2007	2008	Total
Female—*n* (%)	193 (49)	196 (49)	202 (51)	591 (49)
Age group in years—*n* (%):				
18–25	54 (14)	51 (13)	43 (11)	148 (12)
26–35	87 (22)	86 (22)	72 (18)	245 (20)
36–45	83 (21)	78 (20)	91 (23)	252 (21)
46–55	80 (12)	77 (19)	83 (21)	240 (20)
56–65	47 (12)	55 (14)	58 (15)	160 (13)
66+	49 (12)	53 (13)	53 (13)	155 (13)
Employment status—*n* (%):				
Full time	192 (48)	211 (53)	218 (55)	621 (52)
Part time	73 (18)	65 (16)	59 (15)	197 (16)
Retired	65 (16)	75 (19)	76 (19)	216 (18)
Home duties	31 (8)	25 (6)	23 (6)	79 (7)
Unemployed	15 (4)	6 (2)	7 (2)	28 (2)
Studying	13 (3)	16 (4)	11 (3)	40 (3)
Sick/unable	10 (3)	2 (1)	6 (2)	18 (2)
Residence in northern Queensland majority of their life—*n* (%)	264 (66)	250 (63)	203 (51) *	717 (60)
Median years of residence if not majority of their life—nearest full year (interquartile range)	10 (4–14)	10 (4–15)	10 (4–19)	10 (4–15)
Home ownership status—*n* (%)				
Owns/buying	295 (74)	283 (71)	303 (76)	881 (73)
Rents	97 (24)	108 (27)	97 (24)	302 (25)
Other	8 (2)	9 (2)	0 (0)	16 (1)
Number living in household:				
Median (interquartile range)	3 (2–4)	2 (2–4)	3 (2–4)	3 (2–4)

* Difference is statistically significant at a level of 0.01 relative to 2004 and to 2007.

**Table 2:** Bivariate associations with median breeding site scores in northern Queensland—2004, 2007, and 2008.

Characteristic	Median Breeding Site Score (*p*-Value)		
	2004	2007	2008
Gender			
Male	0.82	0.77	0.75
Female	0.73	0.69	0.69
	(0.009)	(<0.001)	(0.008)
Age group (in years)			
18–25	0.82	0.77	0.69
26–35	0.82	0.77	0.69
36–45	0.73	0.73	0.69
46–55	0.81	0.77	0.75
56–65	0.81	0.77	0.72
66+	0.81	0.69	0.69
	(0.443)	(0.451)	(0.677)
Paid employment status			
Working	0.82	0.77	0.75
Not working	0.82	0.69	0.69
	(0.951)	(0.001)	(0.013)
Duration of residence in northern Queensland			
Most of life:			
Yes	0.82	0.77	0.75
No	0.82	0.77	0.69
	(0.553)	(0.726)	(0.591)
Median years of residence if not majority of their life (+/− median of 10 years)			
10 or longer	0.82	0.77	0.75
Less than 10	0.82	0.77	0.69
	(0.622)	(0.579)	(<0.001)
Home ownership status			
Owns/buying	0.81	0.77	0.75
Rents	0.73	0.77	0.69
	(0.125)	(0.693)	(<0.001)
Household size (+/− median of three members)			
Three or more	0.82	0.77	0.69
Two or less	0.82	0.77	0.69
	(0.295)	(0.906)	(0.713)

Note: Mann–Whitney and Kruskal–Wallis tests used where appropriate.

**Table 3:** Independent predictors of median and above breeding site scores—2004, 2007, and 2008.

Model Inclusions *	Score Ratio ** (95% Confidence Intervals)	Difference in Scores *** (95% Confidence Intervals)	*p*-Value
2004 (*n* = 390)			
Male sex	1.18 (0.99–1.42)	0.09 (−0.01–0.19)	0.069
Home ownership	1.28 (1.01–1.62)	0.13 (0.01–0.24)	0.049
2007 (*n* = 391)			
Male sex	1.52 (1.26–1.85)	0.22 (0.12–0.22)	<0.001
Home ownership	0.94 (0.77–1.14)	−0.02 (−0.13–0.10)	0.513
2008 (*n* = 400)			
Male sex	1.34 (1.10–1.65)	0.14 (0.05–0.24)	0.005
Home ownership	1.40 (1.05–1.86)	0.15 (0.04–0.26)	0.02

* All log-binomial models were also adjusted for age; ** ratio of above to below median breeding site score; *** absolute difference in proportions represented in breeding site score group.
